# Association between maximum temperature and PM_2.5_ with pregnancy outcomes in Lima, Peru

**DOI:** 10.1097/EE9.0000000000000179

**Published:** 2021-11-12

**Authors:** Vilma L. Tapia, Bertha Vanessa Vasquez-Apestegui, Diana Alcantara-Zapata, Bryan Vu, Kyle Steenland, Gustavo F. Gonzales

**Affiliations:** a Laboratorio de Endocrinología y Reproducción, LID, Universidad Peruana Cayetano Heredia, Lima, Peru; b Department of Biological and Physiological Sciences, Faculty of Sciences and Philosophy, Universidad Peruana Cayetano Heredia, Lima, Peru; c Environmental Health, Rollins School of Public Health, Emory University, Atlanta, Georgia.

**Keywords:** High temperature, Birth weight, Pregnancy, Preterm birth, Heat

## Abstract

**Background:**

We have previously documented an inverse relationship between PM_2.5_ in Lima, Peru, and reproductive outcomes. Here, we investigate the effect of temperature on birth weight, birth weight-Z-score adjusted for gestational age, low birth weight, and preterm birth. We also explore interactions between PM_2.5_ and temperature.

**Methods:**

We studied 123,034 singleton births in three public hospitals of Lima with temperature and PM_2.5_ during gestation between 2012 and 2016. We used linear, logistic, and Cox regression to estimate associations between temperature during gestation and birth outcomes and explored possible modification of the temperature effect by PM_2.5_.

**Results:**

Exposure to maximum temperature in the last trimester was inversely associated with both birth weight [β: −23.7; 95% confidence interval [CI]: −28.0, −19.5] and z-score weight-for-gestational-age (β: −0.024; 95% CI: −0.029, −0.020) with an interquartile range of 5.32 °C. There was also an increased risk of preterm birth with higher temperature (interquartile range) in the first trimester (hazard ratio: 1.04; 95% CI: 1.001, 1.070). The effect of temperature on birthweight was primarily seen at higher PM_2.5_ levels. There were no statistically significant associations between temperature exposure with low birth weight.

**Conclusions:**

Exposition to maximum temperature was associated with lower birth weight and z-score weight-for-gestational-age and higher risk of preterm birth, in accordance with much of the literature. The effects on birth weight were seen only in the third trimester.

**What this study adds**

High temperature and PM_2.5_ are two environmental factors that have been negatively associated with pregnancy outcomes. This study found that in Lima, a city without extreme seasonal temperatures (annual maximum average is 23.7 °C), the maximum temperature during the third trimester is associated with lower birth weight and a lower z-score weight-for-gestational-age. Furthermore, this association is stronger when PM_2.5_ is higher. Thus, our study contributes with the evidence to show the risk of maximum temperature and high levels of PM_2.5_ on reproductive outcomes, even in a city with annual moderate temperatures.

## Background

The increase in the frequency and intensity of weather events over the last 50 years is emerging evidence generating global health problems.^1^ Heatwave/extreme temperature is related to multiple health problems^2–6^ that can be more pronounced in vulnerable populations such as the elderly,^7,8^ infants,^9,10^ and pregnant women.^11^

Metropolitan Lima is the capital city of Peru, located on the central coast of the country. Lima has a warm temperate climate, with an annual mean temperature of 20 °C. In addition, Lima is one of the most polluted cities in South America,^12^ where the levels of PM_2.5_ and PM_10_ are have often been found over the Peruvian permissible limit (National Annual PM_2.5_: 25 µg/m^3^; PM_10_: 50 µg/m^3^).

Birth weight is a crucial health indicator with significant short- and long-term health implications.^13^ Low birth weight (LBW) and small for gestational age (SGA) are associated with chronic diseases in adulthood.^14,15^ Preterm birth is the major cause of death in children <5 years and is associated with an increased risk of long-term cognitive function impairments.^16^ In Lima, the LBW and PTB rates in 2015 were 5.4% and 6.47%, respectively.^17^

During pregnancy, hormonal and physiological changes occur^18,19^ that could influence maternal thermoregulatory capacity, making pregnant women more vulnerable to heat stress.^20^ For example, extreme temperatures during pregnancy are associated with oxidative stress and systemic inflammation.^21,22^ These conditions could decrease uterine and placental–fetal blood flow, decreasing fetal growth.^23^

Studies have shown an association between heat waves and high temperature with birth weight, LBW, and preterm birth. Chersich et al.^11^ conducted a systematic review and meta-analysis using 70 studies in 27 countries. They found that higher temperature (temperatures above the 75th centile) was associated with reduction of birthweight [−25.5; confidence interval (CI) 95%, −39.4, −15.0] based on six studies, and odds of LBW (<2,500 g) were increased (odds ratio of 1.09 (1.04–1.07) in nine studies. The odds of a preterm birth rose 1.05-fold (1.03–1.07) per 1 °C increase in temperature and 1.16-fold (1.10–1.23) during heatwaves.^11^

A large number of studies have also pointed to air pollution as a factor that is associated with pregnancy outcomes. In the most recent systematic review and meta-analysis, Ghosh et al.,^24^ considered 40 studies with data on PM_2.5_ and birth outcomes, and found 22 g [95% Uncertainty interval (UI): 12, 32] lower birth weight, 11% greater risk of LBW (1.11, 95% UI: 1.07, 1.16), and 12% greater risk of PTB (1.12, 95% UI: 1.06, 1.19), per 10 μg/m^3^ increment in ambient PM_2.5_. Bekkar et al.^25^ reached similar conclusions in a meta-analysis restricted to US studies.

Ghosh et al.^24^ estimate that globally 16% of all LBW babies and 36% of all PTB infants were attributable to total PM_2.5_, with perhaps a third contributed by ambient PM_2.5_, and two-thirds to household air pollution. As Chersich et al.^11^ point out in their review, there are no comparable figures for the risk of adverse birth outcomes attributable to heat, but clearly with climate change, if heat is indeed causally related to adverse birth outcomes, the attributable risk can only increase as temperature increases in the future.

However, exposure to combined effects of temperature and air pollutants on birth outcomes has been scarcely studied.^26,27^ Wang et al.^27^ evaluated heatwaves on PTB and reported synergistic effects of PM_2.5_ and heat waves, with more PTB with a combination of high PM_2.5_ and heatwaves in the week before birth. Kwag et al.,^26^ in contrast, found that PM_2.5_ exposure during the first trimester (TR1) increased the risk of preterm birth among pregnant women exposed to low temperatures during TR1.

There are no studies related to the effect of temperature on pregnancy outcomes in Lima. The present study aimed to investigate the association between maximum temperature exposure with birth weight, birth weight-Z-score adjusted for gestational age, LBW, and PTB, across all of the gestation and by different trimesters, in the metropolitan area of Lima during the period of 2012 to 2016. We also explore possible interactions with PM_2.5_. We have previously published results regarding the effects of air pollution on birth outcomes in Lima, where we found that higher PM_2.5_ was associated with lower birth weight and more risk of being born SGA, although not significantly associated with PTB.^28^

## Methods

### Study design and study area

This study investigates the association of high temperature and PM_2.5_ with pregnancy outcomes in Lima, Peru, from 2012 to 2016. The pregnant women were identified by birth records from Lima hospitals, with exposure to heat and PM_2.5_ ascertained retrospectively before the birth.

Metropolitan Lima is the capital of Peru, located on the country’s central coast at 150 m above sea level with an approximate population of 9,174,855 inhabitants.^29^ This city has a subtropical climate, high humidity, and low rainfall. The average annual temperature is 20 °C with an average maximum and minimum temperature of 25 °C and 17.9 °C, respectively. These conditions give it a pleasant climate without excessive tropical heat in the summer months (29 °C) or extreme cold in winter (14 °C).

Lima comprises 43 districts, which were the basis for assigning exposure to women residents in these districts. From the total population, 28.5% are women of childbearing age, and 2.13% are pregnant women.^29^

### Study population

We obtained data on live births records from the Perinatal Information System (SIP 2000) from one large public hospital (Santa Rosa Hospital) and two exclusive maternal care facilities (San Bartolome hospital and National Maternal and Perinatology Institute). All three are located in the central area of Lima and receive patients from all Lima districts; 26 % of births from Lima occur at the sites included here. The data covers January 2012 to December 2016 and includes information on mothers and their babies. We excluded multiple births (n = 7,430), malformations based on clinical practice guidelines (n = 855), mothers residing in other provinces (466), duplicate records (n = 1,941), stillbirths (n = 1,469), outliers (birth weight ± three SD according to gestational age n = 340).

In addition, we excluded missing data from the district of residence (n = 150), mother’s age (n = 69), and missing PM_2.5_ estimators (n = 3,080, corresponding to the four altitude districts where our model could not reliably estimate PM_2.5_). Our final population sample included 123,034 live newborns of singleton births.

### Outcome variables

Our objective was to study adverse birth outcomes, including lower weight at birth (continuous), birthweight among full-term births (≥37 weeks), and weight-by-gestational age (z-score), as well as LBW (dichotomous) and PTBs (dichotomous). We did not study stillbirths in this study because they will be the subject of another paper.

Birth weight, expressed in grams, was defined as the first weight of the baby obtained from a calibrated scale by trained staff in each of the three hospitals selected for the study. LBW was defined as birth weight less than 2,500 g at 37–42 gestational weeks. PTB was defined by delivery with <37 weeks of gestation. We also studied the birth weight-Z-score adjusted for gestational age (WAZ), determined from INTERGROWTH tables (https://intergrowth21.tghn.org/).

### Meteorological and PM_2.5_ data

We used daily maximum temperature (*T*_max_) estimated at 0.1° gridded spatial resolution from PISCO (Peruvian Interpolated Data of the SENAMHI’s Climatological and Hydrological Observations). This database was provided by SENAMHI (National Weather Service of Meteorology and Hydrology of Peru) (https://www.gob.pe/senamhi). These data were constructed from interpolated data from 684 air temperature monitors throughout the country and remote-sensed data.^30^ We used population-weighted data to derive a daily maximum temperature for Lima as a whole. For each birth, we calculated average temperature across Lima by trimester and entire pregnancy using the date of last menstrual period (LMP) to 13 weeks of gestation for the first trimester, from 14 to 26 weeks for the second trimester and, from 27 weeks to birth for the third trimester.

To evaluate PM_2.5_ exposure, we used an estimated data from a model developed by Vu et al.,^31^ which combined satellite measurements, chemical transport model (WRF-Chem), and ground measurements to predict daily PM_2.5_ at a 1 km2 spatial resolution; the model had an R-square of 0.70 when compared to ground measurements. From this model, we obtained daily PM_2.5_ data by district (n = 43) assigned to each pregnant woman according to her district of residence, averaging across all 1 km^2^ grids in the district for each day. On every 16th day throughout the study period, we could not to estimate PM_2.5_ due to lack of satellite coverage, and we averaged across nonmissing data for those days.

We were unable to estimate PM_2.5_ in four districts (4% of Lima’s population) due to the altitude and the lack of ground monitors there. Besides, we did not want to extrapolate our altitude effect from our predictive model out to very high altitudes. Overall, we had PM_2.5_ estimates for 91% of the days in our study period. When we were missing a day in calculating an average over gestation or over a trimester, we calculated the average ignoring the missing data.

There were no comparable data for other pollutants such as nitrogen dioxide or ozone, where only spare ground monitoring data were available, without a model to estimate daily exposure.

### Covariables

Gestational age at delivery was determined by the LMP. According to Capurro’s methodology, the values missing (n = 105) in the SIP database were replaced by the gestational age determined by physical examination.^32^ Mother’s age was defined as age at delivery (categorized as <20, 20–34, and ≥35 years). Parity was categorized as childless, 1–2, or and, >2 births). Prenatal care was dichotomized as with medical control (≥1) versus without medical control. We also included a variable for the presence of any medical complications during pregnancy, such as preeclampsia and urinary infection. The percentage of poverty was defined as the households within a district with a self-reported family income below what is needed for sustenance, food, clothing, or shelter by the Peruvian Census.^33^ We dichotomized poverty into districts above and below the median poverty level. Smoking was not considered since the rate is very low (<1%) in pregnant women in Peru^34^ and data are not recorded in the database. Because warm days are often more polluted, PM_2.5_ was included as a covariate in birth outcome models, averaged over gestation or by trimester.

## Statistical analysis

Data were collected in an Excel spreadsheet and analyzed using Stata software version 14.0 (STATA Corp, TX). We calculated the population-weighted average of pollutant PM_2.5_ by district and population-average temperature for the entire pregnancy by trimester. We performed analysis for each pregnancy outcome across all gestation periods, and during each trimester, in separate models.

We used multiple linear regression to explore the association between maximum temperature exposures during pregnancy with birth weight as a continuous variable for all births as well as restricted to full-term infants. We also studied the WAZ using a multiple linear regression model. Logistic regression analysis was used to determine the relationship of temperature with LBW.

We used a Cox proportional hazards model to estimate the hazard ratio for PTB by temperature. The time variable days of gestation up to 37 weeks, at which point observations were censored, as no PTBs could occur after that point. In the Cox analysis, each PTB was compared, regarding temperature exposure, to all other non-PTB births up to the time of the PTB event. While it would have been possible to study PTBs as yes/no events across all births via logistic regression, we felt that time-to-event provided increased information and chose a Cox model. We included in the Cox model the time-varying variables temperature and PM_2.5_, averaged entire gestation, or according to trimester, as well as time invariant covariates (see below).

Based on the known determinants that influence birth weight, we included maternal age (<20, 20–34, and ≥35), poverty (no and yes), prenatal care (control and no control), parity (none, 1–2, and >2 births), preeclampsia (no and yes), urinary infection (no and yes) initially in all regression models. Variables that by themselves attained a *P* value < 0.1 were included as candidate covariates, and those that retained statistical significance at *P* < 0.05 were included in the final model. Final models for continuous birthweight and z-score included covariates for maternal age, parity, gestational age, preeclampsia, prenatal care, poverty, for LBW the same variables minus poverty and final models for PTB included mother’s age, preeclampsia, prenatal care, parity, and urinary infection as time-invariant covariate.

We evaluated multicollinearity between the variables included in the regression models using the variance inflation factor (VIF).

As an additional analysis of the average maximum temperature as a linear term, we analyzed quartiles according to all gestation and specific time windows. For the entire pregnancy, the quartiles were: Q1st: 17.65–22.63 °C; Q2nd: 22.64– 23.76 °C: Q3rd: 23.77–24.86 °C, and Q4th: 24.87–29.04 °C. Quartiles were similar for each trimester. The interquartile temperature range (IQR) for the entire pregnancy was 2.23 °C and for trimesters: 5.43 °C, 5.32 °C, and 5.23 °C, respectively, with more variation for IQRs by trimester due to the smaller sample sizes used for calculations. For the analysis, we considered an IQR of 5.32 for all trimesters.

For adjustment by air pollution, we included PM_2.5_ as a continuous variable in all models, and we also explored the interaction temperature-PM_2.5_ using a dichotomous PM_2.5_ level above or below the median (≥18.86 µg/m^3^ vs. <18.86 µg/m^3^) and continuous temperature, during the exposure window (all gestation or trimester). We tested the interaction in the total population and also ran separate models below and above the median.

The protocol was approved by the Ethics Review Committee of Cayetano Heredia University (SIDISI code 101546).

## Results

The present study included 123,034 pregnant women. Of these, 70% were aged 25 to 34 years, 84% had a partner, 12% had high education, 7% had more than two children, and 74% had prenatal care. Among all pregnancies, 8,897 (7.16%) were PTB and, among those with full-term births, 2,074 (1.78%) were LBW (Table 1).

**Table 1.** Descriptive characteristics of mothers and newborns, Lima, Peru, 2012–2016

Characteristic	Number	Frequency (%)
Mothers age (years)		
<20	16,853	13.7
20–34	84,998	69.1
>34	21,183	17.2
Pre-eclampsia (%)		
No	117,813	95.8
Yes	5,221	4.2
Urinary infection (%)		
No	112,045	91.1
Yes	10,803	8.8
Missing	186	0.1
Parity		
0	58,951	47.9
1–2	52,151	42.4
>2	9,364	7.6
Missing	2,568	2.1
Poverty (%)		
No	61,790	50.2
Yes	61,244	49.8
Prenatal care (%)		
No control	24,171	19.7
Control	95,899	77.9
Missing	2,964	2.4
Preterm birth (%)		
No	114,137	92.8
Yes	8,897	7.2

Descriptive statistics for temperature, PM_2.5_, birth weight, and z-score for birth weight are shown in Table 2.

**Table 2.** Environmental Characteristic and Birth Weight

Characteristic	Mean ± sd	P25	P50	P75	P90
Maximum temperature (°C)	23.7 ± 1.5	22.6	23.8	24.9	25.6
PM2.5 (µg/m^3^)	21.2 ± 5.3	16.8	18.4	26.0	28.7
Birth weight (g)	3,355 ± 554	3,080	3,390	3,696	3,980
Birth weight at term (g)	3,434 ± 449	3,144	3,430	3,720	4,000
Z-score W/GA	0.261 ± 1.14	−0.44	0.25	0.95	1.65

P indicates percentile; Sd, standard deviation.

We evaluated the relationship between birth weight and temperature (Table 3), with results for the entire gestation and trimester of gestation. While there was no marked reduction in birthweight with higher temperature across all gestation, we observed a reduction of 23 g (95% CI: −28.0, −19.5; R^2^: 0.66, *P* < 0.0001) in birth weight per increase an IQR (5.32 °C) of maximum temperature in the third trimester. Evaluated by quartiles, there was a monotonic decrease in birthweight with higher quartiles of temperature. The highest quartile of temperature (>26.0 °C) was associated with the largest reduction in birth weight (β: −30.6 g; 95% CI: −37.67, −23.59). In contrast, we observed a positive relationship between temperature and birthweight in the first trimester (linear model, β: 16.1 g; 95% CI 12.0; 20.2).

**Table 3.** Relationship Between Maximum Temperature (IQR*) With Birth Weight and Z-score Weight by Age During the Pregnancy, 2012–2016

Pregnancy	Birth Weight B*IQR (95% CI)	Weight Z-score by Age B* IQR (95% CI)
Entire pregnancy: Max temperature (°C)	−3.19 (−7.180, 0.796)	−0.01 (−0.020, −0.001)
Max temperature quartiles:		
Q1st (17.66–22.63)	1.0	1.0
Q2nd (22.64–23.76)	0.44 (−6.588, 7.483)	0.02 (0.007, 0.043)
Q3rd (23.77–24.86)	1.47 (−5.714, 8.669)	0.02 (0.001, 0.038)
Q4th (24.87–29.04)	−8.11 (−16.02, −0.196)	−0.01 (−0.035, 0.005)
1st trimester:		
Max temperature (°C)	16.1 (12.02, 20.21)	0.01 (0.009, 0.018)
Max temperature quartiles:		
Q1st (16.64–20.76)	1.0	1.0
Q2nd (20.77–23.11)	7.10 (0.151, 14.06)	0.01 (−0.011. 0.024)
Q3rd (23.12–26.19)	15.3 (8.339, 27.26)	0.03 (0.015, 0.051)
Q4th (26.20–32.44)	27.2 (20.05, 34.36)	0.05 (0.034, 0.071)
2nd trimester		
Max temperature (°C)	−0.16 (−4.394, 6.083)	−0.002 (−0.013, 0.003)
Max temperature quartiles:		
Q1st (16.64–20.76)	1.0	1.0
Q2nd (20.77–23.11)	−7.19 (−14.12, −0.262)	−0.02 (−0.035, 0.001)
Q3rd (23.12–26.19)	−6.64 (−13.54, 0.273)	−0.01 (−0.035, 0.0001)
Q4th (26.20–32.44)	0.73 (−6.364, 7.843)	0.001 (−0.017, 0.019)
3rd trimester		
Max temperature (°C)	−23.7 (−28.03, −19.52)	−0.02 (−0.029, −0.020)
Max temperature quartiles		
Q1st (16.64–20.76)	1.0	1.0
Q2nd (20.77–23.11)	−7.89 (−14.80, −0.979)	−0.01 (−0.034, 0.001)
Q3rd (23.12–26.19)	−26.2 (−33.16, −19.40)	−0.04 (−0.062, −0.026)
Q4th (26.20–32.44)	−30.6 (−37.67, −23.59)	−0.07 (−0.097, −0.061)

Results in bold are significant at *P* < 0.05.

Effects estimated change per IQR for max temperature. IQR max temperature: 2.23 °C by entire pregnancy and IQR: 5.32°C by trimesters; Birth weight model adjusted by PM_2.5_, mother’s age, preeclampsia, parity, prenatal care, poverty, and gestational age. Z-score model adjusted by the same variables, except gestational age.

Β indicates coefficient; C, centigrade; CI, confidence interval; IQR, interquartile range.

Analyzing the weight z-score by age, we found a significant inverse relationship during all gestation, more marked in third trimester of pregnancy (β: −0.02; 95% CI: −0.029, −0.020) and a monotonic inverse relationship by quartiles during the third trimester. Again, in contrast, there was a positive trend in z-score during the first trimester.

When we restricted the analysis to full-term babies, we found a similar pattern to the estimations observed in the total population, meaning a decrease of 25 g in the third trimester per temperature IQR, and, in contrast, an increase of 18 g in birth weight per IQR of temperature in the first trimester (Table 4). For PTB, we found a slightly increased risk of PTB per IQR increase in temperature during the first (hazard ratio: 1.04, 95% CI: 1.001, 1.07) and second (hazard ratio: 1.04; 95% CI: 1.005, 1.08) trimesters (Table 4).

**Table 4.** Association Between Maximum temperature with term birth weight and PTB During the Pregnancy, 2012–2016

Pregnancy	Term Birth Weight B*IQR (95% CI)	Preterm Birth HR exp(B*IQR)) (95% CI)
Entire pregnancy		
Max temperature (°C)	−1.27 (−5.37, 2.83)	1.04 (1.01, 1.07)
Max temperature quartiles		
Q1st (17.66–22.63)	1.0	1.0
Q2nd (22.64–23.76)	0.55 (−6.565, 7.671)	0.68 (0.646, 0.731)
Q3rd (23.77–24.86)	0.66 (−6.651, 7.972)	0.78 (0.734, 0.830)
Q4th (24.87–29.04)	−6.09 (−14.0, 2.022)	0.96 (0.906, 1.032)
1st trimester		
Max temperature (°C)	18.3 (14.20, 22.45)	1.04 (1.001, 1.07)
Max temperature quartiles		
Q1st (16.64–20.76)	1.0	1.0
Q2nd (20.77–23.11)	10.3 (3.67, 17.33)	0.97 (0.921, 1.039)
Q3rd (23.12–26.19)	19.1 (12.11, 26.20)	0.97 (0.915, 1.032)
Q4th (26.20–32.44)	30.1 (2.86, 37.34)	1.06 (1.006, 1.136)
2nd trimester		
Max temperature (°C)	1.43 (−2.819, 5.69)	1.04 (1.005, 1.08)
Max temperature quartiles		
Q1st (16.64–20.76)	1.0	1.0
Q2nd (20.77–23.11)	−4.8 (−11.82, 2.171)	1.01 (0.956, 1.079)
Q3rd (23.12–26.19)	−3.75 (−10.74, 3.239)	1.12 (1.061, 1.194)
Q4th (26.20–32.44)	3.76 (−3.421, 10.95)	1.04 (0.980, 1.108)
3rd trimester		
Max temperature (°C)	−24.8 (−29.20, −20.58)	0.99 (0.95, 1.02)
Max temperature quartiles		
Q1st (16.64–20.76)	1.0	1.0
Q2nd (20.77–23.11)	−8.68 (−15.62, −1.682)	0.97 (0.915, 1.029)
Q3rd (23.12–26.19)	−28.6 (−35.56, −21.68)	0.80 (0.931, 1.065)
Q4th (26.20–32.44)	−33.0 (−40.14, −25.86)	1.02 (0.966, 1.088)

Bolder estimates are significant a *P* < 0.05.

Effect estimated presented as Β per IQR max temperature. IQR max temperature: 2.23 °C by pregnancy and IQR: 5.32°C by trimesters; Regression Model adjusted by age, preeclampsia, parity, PM_2.5_, prenatal care, poverty, and gestational age. Cox regression model was adjusted by age, PM_2.5_, preeclampsia, prenatal care, parity, and urinary infection.

Β indicates coefficient; C, centigrade; CI, confidence interval; HR, hazard ratio; IQR, interquartile range.

No significant associations (Table 5) were observed between exposure to temperature and LBW.

**Table 5.** Association Between Maximum Temperature With LBW During the Pregnancy, 2012–2016

Pregnancy	Low Birthweight OR = β * IQR (95% CI)
Entire pregnancy	
Max temperature (°C)	1.00 (0.93, 1.07)
Max temperature quartiles:	
Q1st (17.66–22.63)	1.0
Q2nd (22.64–23.76)	0.87 (0.76, 1.01)
Q3rd (23.77–24.86)	0.99 (0.87, 1.13)
Q4th (24.87–29.04)	1.01 (0.87, 1.16)
1st trimester	
Max temperature (°C)	0.98 (0.91, 1.01)
Max temperatura quartiles:	
Q1st (16.64–20.76)	1.0
Q2nd (20.77–23.11)	0.93 (0.82, 1.06)
Q3rd (23.12–26.19)	1.02 (0.90, 1.15)
Q4th (26.20–32.44)	0.95 (0.83, 1.08)
2nd trimester	
Max temperatura (°C)	1.01 (0.93, 1.08)
Max temperature quartiles	
Q1st (16.64–20.76)	1.0
Q2nd (20.77–23.11)	1.05 (0.93, 1.19)
Q3rd (23.12–26.19)	1.09 (0.97, .24)
Q4th (26.20–32.44)	1.01 (0.88, 1.15)
3rd trimester	
Max temperature (°C)	1.03 (0.95, 1.11)
Max temperature quartiles	
Q1st (16.64–20.76)	1.0
Q2nd (20.77–23.11)	0.97 (0.85, 1.09)
Q3rd (23.12–26.19)	1.01 (0.89, 1.14)
Q4th (26.20–32.44)	1.02 (0.90, 1.16)

IQR (2.23 °C) for entire pregnancy. IQR: 5.32 °C for the trimesters. Logistic regression model adjusted by age, preeclampsia, prenatal care, gestational age, and parity.

Β indicates coefficient; CI, confidence interval; IQR, interquartile range; OR, odd ratios.

We explored modification by PM_2.5_ of the effect of temperature on birth outcomes by including an interaction term for the continuous variable temperature and PM_2.5_ and stratifying our analyses of temperature and birth outcomes by high and low PM_2.5._ (Table 6). A significant interaction term between temperature and PM_2.5_ was observed for birth weight in the entire pregnancy (*P* = 0.03) and third trimester (*P* = 0.002), with the effect of temperature on birthweight stronger with higher PM_2.5_. Similar patterns were observed for weight-for-age z-score and full-term infants. For LBW, we found a significant increased risk with higher PM_2.5_, but only in the third trimester.

**Table 6.** Relationship Between Temperature and Birth Outcomes Stratified by High or Low PM_2.5_, 2012 to 2016

Outcome	Exposure	PM2.5 Level	B*IQR for Temperature	95% CI	*P* for Interaction Between Continuous PM2.5 and Temperature
Birth weight	Entire pregnancy	Low	0.69	−4.72, 6.13	0.027
		High	−10.2	−16.2, −4.10	
	1st trimester	Low	14.7	8.99, 20.6	0.340
		High	20.9	15.0, 26.8	
	2nd trimester	Low	3.7	−2.18, 9.73	0.337
		High	−3.13	−9.5, 2.92	
	3rd trimester	Low	−17.9	−23.9, −12.0	0.002
		High	−28.7	−34.8, −22.6	
Weight Zscore	Entire pregnancy	Low	−0.006	−0.02, 0.004	0.01
		High	−0.01	−0.03, −0.001	
	1st trimester	Low	0.015	0.004, 0.031	0.079
		High	0.05	0.04, 0.06	
	2nd trimester	Low	0.003	−0.01, 0.01	0.245
		High	−0.00	−0.015, 0.010	
	3rd trimester	Low	−0.04	−0.05, −0.03	0.006
		High	−0.06	−0.07, −0.04	
Birth Weight at term	Entire pregnancy	Low	1.89	−3.65, 7.49	0.043
		High	−8.13	−14.4, −1.82	
	1st trimester	Low	−16.2	10.3, 22.0	0.115
		High	23.6	17.6, 29.6	
	2nd trimester	Low	5.10	−0.0, 11.1	0.330
		High	−1.48	−7.66, 4.62	
	3rd trimester	Low	−19.6	−25.6, −13.5	0.007
		High	−42.9	−35.5, −3.10	
Low birth weight (OR)	Entire pregnancy	Low	0.97	0.88, 1.06	0.411
		High	1.09	0.97, 1.22	
	1st trimester	Low	1.02	0.92, 1.12	0.091
		High	0.94	0.84, 1.04	
	2nd trimester	Low	0.98	0.88, 1.09	0.819
		High	1.03	0.93, 1.15	
	3rd trimester	Low	0.96	0.86, 1.07	0.013
		High	1.13	1.02, 1.27	
Preterm birth (HR)	Entire pregnancy	Low	1.11	1.03, 1.07	0.001
		High	0.90	0.85, 0.95	
	1st trimester	Low	1.12	1.07, 1.17	0.001
		High	0.99	0.95, 1.00	
	2nd trimester	Low	1.11	1.05, 1.16	0.006
		High	0.97	0.92, 1.02	
	3rd trimester	Low	1.08	1.03, 1.14	0.001
		High	0.88	0.84, 0.99	

Bolded estimates are significant a *P* < 0.05.

IQR (2.23 °C) for entire pregnancy. IQR: 5.32°C for the trimesters. Linear regression model adjusted by age, preeclampsia, prenatal care, parity, poverty, and GA. Cox model for preterm birth adjusted by age, preeclampsia, parity, prenatal care, and urinary infection. High PM_2.5_ is above the median, and Low is at or below the median.

β indicates coefficient; CI, confidence interval; HR, hazard ratio; IQR, interquartile range.

## Discussion

This study is focused on evaluating the relationship between maternal exposure to maximum temperature and PM_2.5_ with birth weight and PTB. Our results showed an inverse association between temperature and birth weight and weight-for-age z-score, more pronounced in the third trimester but no association with LBW. We found an association between higher temperature and PTB, but in contrast to birth weight, this association was seen in the first and second trimesters but not the third.

Our results are consistent with most studies in the literature which found that higher temperatures were associated with lower birth weight.^11^ The effect of maximum temperature on birth weight during the last trimester could be explained by the faster growth of the fetus in this period, which might be a time of increased susceptibility. Exposure to high temperatures could decrease uterine blood flow and restricting the fetal growth.^21^

In contrast, we found an increase in birth weight associated with temperature in the first trimester. There is some evidence that heat stress could cause a greater degree of hypoxemia in immature placental tissue, triggering the expression of vascular endothelial growth factor (VEGF) as a response. This activation can promote branching angiogenesis, reversing the placental hypoxia status,^35,36^ and improving the growth of the fetus. Furthermore, infants whose mothers were exposed to peak sunshine during their first trimester were born significantly heavier than their counterparts with such exposure. Sunshine during early gestation might increase the level of insulin-like growth factor (IGF-1), facilitating prenatal growth.^37^

Regarding LBW, most studies have shown an independent association with temperature, while we did not.

We found a slightly increased risk of PTB per IQR increase in temperature, primarily during the first trimester. Several studies have reported that exposure to high-temperature changes increases the risk of premature birth.^26,38^ Heat stress could activate the fetal hypothalamic-pituitary-adrenal axis, prompting the placental release of estriol, prostaglandins which could induce labor onset.^38^

Evaluating a modifying effect by PM_2.5_, our results showed that the negative effect of temperature on the birth weight (as well as low birthweight) was primarily seen at higher values of PM_2.5_, particularly in the third trimester. At the same time, the positive effect of temperature in the first trimester was also slightly accentuated by higher PM_2.5._ In contrast, when we analyzed the interaction between PM_2.5_ and temperature for PTB, we found that the risk of PTB was higher during low exposure to PM_2.5_, but the effects were not strong. The most significant fetal growth occurs during the third trimester when the fetus is vulnerable to any harmful stimulus. Concerning PTB, we have no explanations for the reverse finding of increased risk of PTB due to heat when PM_2.5_ levels are low.

One strength of our study is temperature data estimated from an extensive new government database (PISCO).^30^ These data were constructed using interpolation techniques that included temperature data from fixed monitoring, satellite data, and geographical features, supplementing available data from two ground monitors. In addition, we used the perinatal records of three large public hospitals located in the city center, which receive pregnant women from all the districts in Lima. One limitation of our data is that our exposure estimates were restricted to PM_2.5_. We had very limited data on other pollutants, while our data on daily PM_2.5_ were complete both temporally and spatially for the entire study period. Another limitation of this study is the lack of data related to the women’s occupation and occupation location. However, this would not be expected to change temperature exposure greatly, as the temperature is distributed relatively uniformly across Lima. Also, we were missing data for some possible confounders such as maternal body mass index, education and nutritional status, conditions that may affect the development of the fetus.^39^ However, it is not clear that these variables would be related to temperature, required to act as confounders. Finally, it is possible that the approximately 25% of births in Lima studied here are not representative of all births in Lima; however, we believe the underlying mechanisms of the effect of heat on birth outcomes are likely to be shared among all births in Lima.

## Conclusion

Maternal exposure to high ambient temperature was associated with reduced birth weight during the third trimester of pregnancy and a slight risk of PTB in the first two trimesters. Our findings suggest that maternal exposure to high concentrations of PM_2.5_ could enhance the effect of temperature on birth weight.

## ACKNOWLEDGMENTS

The authors thank to the Ministry of Health (MoH), the hospital directives and SENAMHI for providing the data for the analysis.

The authors declare that they have no conflicts of interest with regard to the content of this report.
